# Book Reviews

**Published:** 1833-04

**Authors:** 


### BIBLIOGRAPHICAL NOTICES.

*A Treatise on Indigestion and its Consequences, called Nervous and Bilious Complaints; with Observations on the Organic Diseases in which they sometimes terminate.* By A. P. W. Philip, M.D., F.R.S. L. and E., &c. Seventh Edition.—8vo. pp. 231. Renshaw and Rush, London.

Elaborate criticism, or a detailed analysis, of a work which bears upon its title-page the verdict which has been passed upon it by the profession, in the two words "Seventh Edition," would be quite supererogatory. In several numbers of this Journal we have given ample accounts of the previous editions of this work, and pointed out the strong claims it possesses to the attention of the student and practitioner. With every wish, therefore, to do all the justice in our power both to the author and the publisher, we confine ourselves to stating, in the words of the former, the additional advantages which will be secured by the purchase of this edition.

"In the present and sixth editions, which are much enlarged, compared with the former editions of this treatise, the more protracted cases of indigestion in particular, both with respect to their symptoms and treatment, are more fully considered. I have endeavoured to trace the causes which render them protracted; to lay down at greater length the plans of treatment which I have found most successful in them, and to ascertain the principles on which their success depends; and a chapter on Symptomatic Indigestion has been added, which is necessary in order to place in a clear point of view the nature of many, particularly of the more protracted cases. In enlarging the following treatise, I have adhered to the plan on which it was originally written, stating merely my own observations, and the reflections suggested by them, and that only when they have led to something which has not been stated or sufficiently explained by others."

Although we are perfectly aware of the great practical merit of some other works on the same subject, we still believe that he who does not peruse Dr. Philip's "Treatise on Indigestion" neither does justice to himself nor to the dyspeptic patients he may have to treat.

---

*Principles of Physiological Medicine, in the form of Propositions; embracing Physiology, Pathology, and Therapeutics, with Commentaries on those relating to Pathology.* By F. J. V. Broussais, M.D. &c. Translated from the French, by Isaac Hays, M.D., and R. Eglesfeld Griffith, M.D., Members of the American Philosophical Society, &c.—Philadelphia: Carey and Lea. 8vo. pp. 594.

We recommend this work to the attention of those who are not sufficiently conversant with the French language to study the original. It is very true that Broussais frequently pushes his peculiar doctrines to an unjustifiable extent; and it is equally certain that he as often lays down propositions, with an assumption of originality, which no well-informed medical student of the present day would deny. Notwithstanding, however, M. Broussais' excessive vanity, which almost every page of his works betrays, he is both an amusing and an instructive writer. His doctrines, we are aware, are no longer so popular in France as they were when they were first promulgated, but they are still supported and acted upon by many very distinguished French practitioners. His opinions upon physiology and pathology are treated with respect even by those who refuse the unlimited belief which M. Broussais considers to be his due: they are frequently referred to by continental and English medical writers, and, for this reason alone, they cannot with propriety be neglected by the student or practitioner who wishes to keep upon a level with the medical literature of the day.

The American translation possesses the great advantage of having a very copious alphabetical index, by the assistance of which M. Broussais' opinions may be instantly referred to.

*An Explanation of the Causes why Vaccination has sometimes failed to prevent Smallpox; and also a Description of a Method, confirmed by experience, of obviating such Causes.* By Edward Leese, Member of the Royal College of Surgeons; Stationary Vaccinator in the National Vaccine Establishment, &c. Part Second.—8vo. pp. 36. London.

Mr. Leese has for many years held the appointment of stationary vaccinator in the National Vaccine Establishment, and must consequently have had sufficient opportunities of becoming well acquainted with every fact appertaining to the subject of vaccination. It is true, indeed, that the best opportunities are not always taken advantage of; the lessons which experience should teach us are too frequently slighted with schoolboy indifference, and neither add one single grain to the "intellectual granary" of the observer, nor enable him to afford one iota of improvement to others. Mr. Leese is not of this careless class: he has carefully attended to every circumstance relating to vaccination, and in the little *brochure* before us he gives, in a brief form, the conclusions he has drawn from the large share of experience he has had.

The "method" referred to in the titlepage is that which was brought into general notice by Mr. Bryce, of Edinburgh.

We doubt whether all Mr. Leese's opinions will be received as undisputed axioms, but the source from whence they proceed will at least secure for them respect and mature consideration. There is one point connected with vaccination, upon which we should be glad to receive information: we allude to the effects of temperature on the vesicle. As far as we know, Dr. Howison, vaccinator to the Royal Dispensary at Edinburgh, is the only person who has recorded his opinion upon this subject: he says, "From long experience, I am inclined to think that temperature, or weather, affects, to a considerable extent, the perfection of the vaccine vesicle. I have always observed that the vesicle is more complete, and more certain of going through its various stages, during the summer months, and particularly during warm seasons, when it is turgid with pellucid veins, than during the winter, when the weather is cold and tempestuous; at which period the vesicle is small, imperfect, and flaccid."*

* Medical Gazette, vol. viii. p. 520. Mr. Leese himself informs us that he is convinced, from his own observation, of the truth of Dr. Howison's remarks. —Rev.

